# Crown and Bridge Work

**Published:** 1920-08

**Authors:** F. W. Frahm


### CROWN AND BRIDGE WORK

BY F. W. FRAHM, A.B., PH.G., D.D.S.

Bridge work as an art dates back to the early centuries. Evidence of this class of work has been gathered to show that this form of restorative dentistry did exist as early as 400 to 500 B. C. The appliances then used were of the simplest form, consisting of gold bands around the remaining teeth, supplying the missing teeth with carvings from ivory or similar material. The historian, Guerini, states that some of these bridge specimens may be seen in the museums of Rome and Athens. There was very little or no real progress made in this art until the beginning of the eighteenth century. However, crown and bridge work, as we know it at present, was not practiced to any extent prior to 1870. At that time Dr. Beers, with the assistance of Dr. Richmond, laid the foundation for the system that has held the profession and public in its golden grip until 1910. It is needless to say that both the profession and public were very willing to be held captive in this embrace.

A great deal of light has been thrown upon this system, greatly to our embarrassment, revealing a condition of affairs that no self-respecting professional man could countenance and remain true to himself. Much of the difficulty thus revealed could be overcome by improvement in the technic of construction and application. But, unfortunately, this future of the work involves only a part of the field; the remaining problems are the most difficult to solve. At this time the difficulties in the technic of construction have resolved themselves into a question of which of the several technics we shall follow or use, regardless of physics or dynamics involved in the case.

In the past, and even into the present, crown and bridge work have been largely mechanical in application and practice. Our thought has ever been that of supplying the lost units of the arches without any evident regard for the laws of function and anatomical requirements. The operator's aim and goal was that of closing up the spaces without a gross violation of the esthetics demanded by the position of the bridge and the occlusion it would have to meet. However, a kindly word for the operator at this time will not be amiss. It must not be forgotten that the conditions surrounding the case and the foundation upon which he was asked to rear a superstructure were not of his own choosing. True, he had the privilege of rejecting the case and refusing to perform the bridging operation until conditions were to his liking, if such a thing was possible. However, we must not forget that the very condition which calls for a crown or bridge does not improve with waiting, but becomes more and more difficult with every day of postponement. A series of pathological conditions could develop and become chronic to a degree that would make such an injury, due to this neglect, quite permanent.

In the training of the student and in the thought of the practitioner, the subject of the physiological function of the teeth, their contours and investing tissues has been sadly neglected. However, I would not go to the length that Dr. R. Ottolengui does and say that bridge workers who are merely mechanics should no longer be permitted to practice. The question of what shall constitute real crown and bridge service is easily a debatable one. Very much can be said for and against either side of it. It is also well to remember that our present-day methods are far too embryonic in character for us to parade them as standards. Should there be a classification of crown and bridge work on the basis of the rich and poor? Should we practice a different system for the middle class, or is it really possible to meet the crown and bridge needs of these three divisions of society with the same system? Is there any justification for making a fixed bridge with a crown at each end for abutment, or may we assume that such a method is now obsolete?

How shall we organize and put over an educational program for both practitioner and patient to help each to see the real value in crown and bridge work. The public at present is very much in favor of fixed bridge work. They are, to some extent, familiar with its shortcomings, but are not willing to lay it aside for something unproven. To explain to the majority of our patients the physics that are involved in each class of bridge work would be analogous to the tale of "water on a duck's back." It is equally difficult to talk with the majority of our patients about tooth movement and its physiology. These functions to their mind are matters far too small for them to consider. What they want is something that will masticate with the least effort and attention on their part.

The practitioner is at present in a crown and bridge imbroglio. He does not know which horn of the dilemma to grasp, both are hedged about with many embarrassing conditions. He makes his appeal to the leaders and teachers, with a hope that they may give him a foundation upon which to stand or help him to loosen the Gordian's knot that seems to hold him. I think I can hear some one say: "Why use any bridge work at all when partial dentures may be used in any case where two or more units are to be supplied?" When such thoughts arise in your mind consult the patient and see what a *crux pons asinorum* you have on hand.

In presenting this series of articles on this branch of practice, I do not expect to lose the *dignus vindice noduce,* for I am only a small unit in our organization. But I shall try and marshal the several units of value in a helpful manner that will not offend a kindly and considerate nature. However, we must not forget that every case of crown and bridge work, either fixed or removable, is not one of our own selection, but a condition forced upon us by a violation of some of nature's laws, or a neglect of her demands.

It would be unprofessional to classify bridge work on the basis of finance. It is unfair and unprofessional to make a removable bridge for one patient because he is a bank director or manufacturer who is abundantly able to pay, and refuse to make a similar one for the minister of the Gospel and college professor, because they do not even possess a bank account and are unable to pay the fee. Are the minister and professor less appreciative and worthy than the bank official? Is the minister's physical resistance higher than that of the man of wealth, so that he can live and thrive with less masticating units than his wealthy fellows? Most assuredly, no! Yet the minister and professor are sent to the exodontist, because they can not afford to have the crown and bridge work done, and the dentist can not afford to lower the standards of technic or fee as they exist today. With each change in practice came an added technic and cost of time and money to the practitioner. He has watched these changes with an aching and heavy heart, because they widen the gulf that is rapidly separating his patients into two classes.—*Pacific Dental Gazette.*

